# Distribution of human papillomavirus infection: a population-based study of cervical samples from Jiangsu Province

**DOI:** 10.1186/s12985-019-1175-z

**Published:** 2019-05-20

**Authors:** C. Zhang, Wj Cheng, Q. Liu, Q. Guan, Qw Zhang

**Affiliations:** 1 The First Affiliated Hospital of Nanjing Medical University, Nanjing, China; 2 Jiangsu Province Hospital of Chinese Medicine, Nanjing, China

## Abstract

**Background:**

Human papillomavirus (HPV) infection may lead to a series of lesions in the cervix. Distributions of HPV genotypes reveal that an increased prevalence of high-risk HPV (HR-HPV) is positively correlated with the severity of cervical lesions. Furthermore, persistent infection of HR-HPV is associated with a risk of cervical cancer. Considering the newly approval of the HPV vaccine in China and the prevalence of HPV distribution, which is meaningful for directing efforts for HPV vaccination, a more detailed understanding of HPV distribution is critical. This study aimed to investigate the overall prevalence of HPV and the age-specific features related to HPV distribution in the Jiangsu population.

**Methods:**

We collected a total of 62,317 cervical cytological specimens from Xuzhou, Nanjing and Suzhou, which represent the northern, middle and southern regions of Jiangsu Province, respectively. All these samples were assigned to 6 groups based on participant age. HPV genotypes tests were performed by using a commercial kit which is designed for the detection of 17 high-risk HPV genotypes and 6 low-risk HPV genotypes.

**Results:**

The overall prevalence of HPV was up to 26.92% in Jiangsu Province. The most common high-risk genotype was HPV52 (5.09%), followed by HPV16, HPV58, HPV53, HPV51 and HPV68. The most prevalent low-risk genotype was HPV81 (2.70%), followed by HPV43, HPV42, HPV6, HPV11 and HPV83. Most infections were caused by HR-HPV, while single-genotype infection occurred more frequently than multiple-genotype infection. Regarding participant age, the overall infection rate of HPV was distributed in a U-shaped manner, with the highest peak in the younger than 20-year-old cohort. Additionally, significant variations were found between different cities, representing different regions of Jiangsu.

**Conclusions:**

HPV prevalence is high in Jiangsu Province. The prevention of HPV-related diseases is challenging. Given the variation in HPV prevalence between ages groups and regions, a flexible HPV vaccination program, adjusted base on regional infection features, could have a beneficial effect in Jiangsu Province.

## Introduction

The strong correlation between cervical HPV infection and cervical lesions or cervical cancer has been well established. HPV infection is the most common sexually transmitted infection. More than 200 HPV genotypes have been identified, with approximately 40 distinct HPV genotypes affecting the mucosal epithelium of the genital tract. HPV genotypes are typically divided into two groups according to their carcinogenicity: the high-risk group (HR-HPV) and the low-risk group (LR-HPV) [1]. Distributions of HPV genotypes reveal that an increased prevalence of HR-HPV is correlated with the severity of cervical lesions [2]. Furthermore, the persistent infection of HR-HPV is associated with a risk of cervical cancer [3, 4]. There was a high incidence and motality of cervical cancer in China [5]. It is a big challenge for the prevention of cervical cancer in China. Cervical cytology combined with HR-HPV testing in married women are the practical methods for detecting cervical lesion and cervical cancer currently in China. National cervical cancer screening guidelines based on demographic and geographic factors and comprehensive implementation strategies are needed in China [6]. In eastern high resource regions, vaccination aganist HPV infection become an acceptable strategy. HPV16 and HPV18, which are the main targets of the current vaccine, contribute to the majority of cervical cancer, as well as to a substantial proportion of cervical intraepithelial neoplasia (CIN) diagnoses of grades 2/3 or higher. HPV6 and HPV11 are the major LR-HPV genotypes that cause genital warts. HPV-related diseases remain a significant cause of morbidity and mortality in developing and developed nations, underscoring the need for HPV vaccination programs with wide coverage. The preponderant burden of HPV16/18 along with the potential cross-protection from other HPV genotypes, emphasizes the importance introducing more affordable vaccines in less-developed countries [7]. Prophylactic HPV vaccines in widespread use include the bivalent (Cervarix, GSK) and quadrivalent vaccines (Gardasil4, Merck). The 9-valent vaccine (Gardasil9, Merck) has recently been approved in several countries, including China. Typically, it has been suggested that an HPV vaccine be administered before the first sexual behavior, such as between the ages of 9–25. However, in women 24 to 45 years of age participating in an international double-blind clinical trial, quadrivalent vaccines (Gardasil4, Merck) have demonstrated efficacy of against the combined incidence of persistent infection and cervical intraepithelial neoplasia [8]. To effectively prevent HPV infection in Jiangsu Province, it is essential to have the accurate data regarding HPV prevalence across different cities and different age groups. The data could be used to ensure the vaccine administration is providing precise protection unique to the specific needs the local population.

**Fig. 1:**
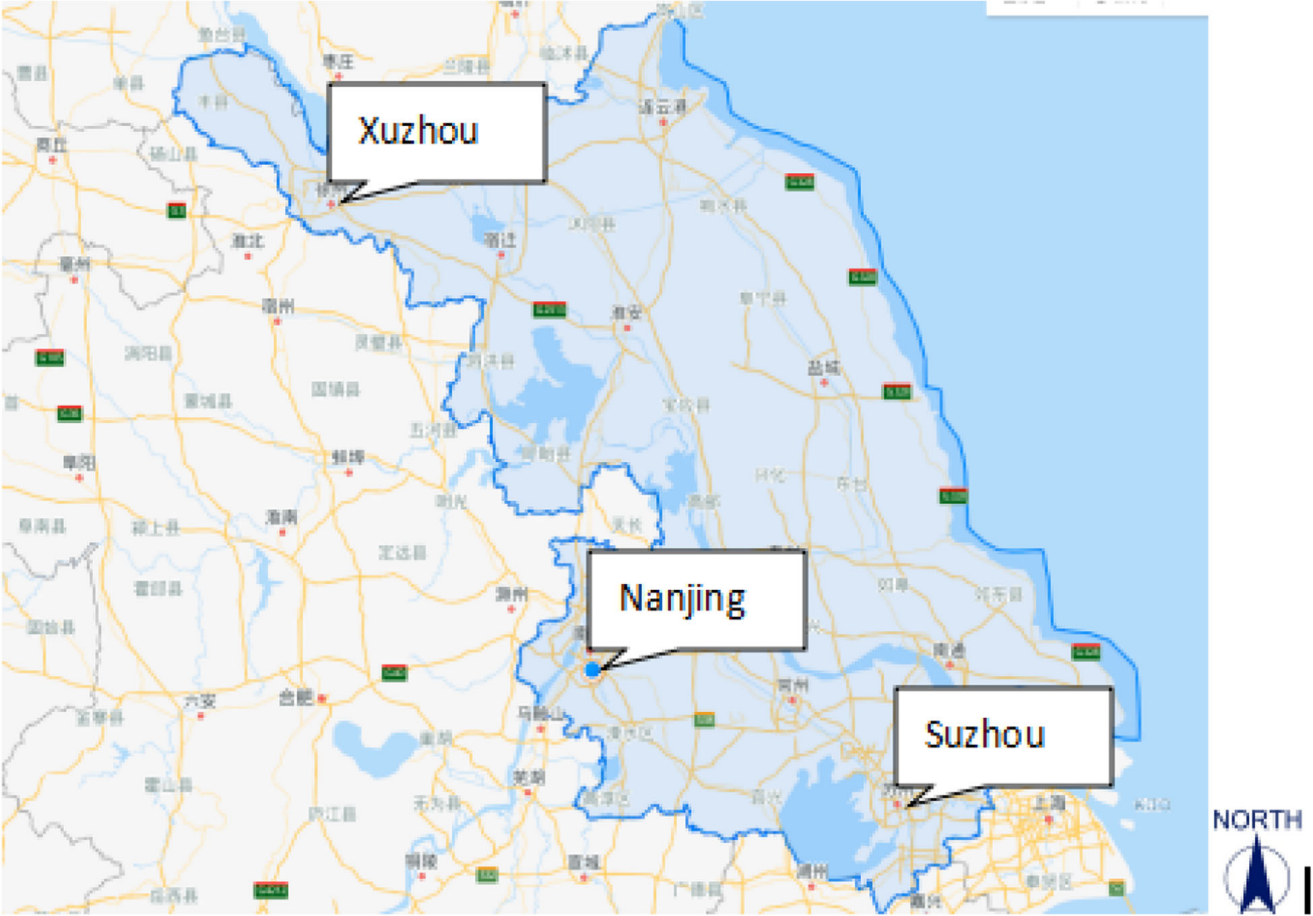
Location of 3 cities in Jiangsu Province

Following the release of the Gardasil 4 HPV vaccine in mainland China, prevention of cervical cancer became widely discussed among the public. HPV vaccine administration in subjects between 26 and 45 years of age has shown safe, effective protection against vaccine-related genital warts and cervical dysplasia [9]. The prevalence of HPV in the Jiangsu population and the age-specific infection rate are very important pieces of data that could inform policy-making regarding local vaccination programs. Three typical cities, representing different regions of Jiangsu, were chosen to describe the geographical prevalence profile of HPV. The map in Fig. 1 pinpoints the three central cities, which lie in separate regions in the north, middle and south of Jiangsu. The three cities are representative of Jiangsu geographically, economically and culturally. We investigated the overall HPV infection rate along with the type-specific infection rate in the three cities. Also the differences of HPV infection between three regions and six age groups were compared. The prevalence data can be used to make a cost-effective HPV vaccine recommendations specific to each city/region.

## Materials and methods

From April 2013 to August 2017, 62,883 cervical samples were collected from women attending routine outpatient gynecological examinations in three cities in Jiangsu Province. For this study, the total of 62,317 cervical samples were acquired from the three included cities regions, and the overall infection rate along with the type-specific infection rate was calculated. The prevalence of HPV infection among different regions and different age groups was compared. Participants were enrolled based on the following criteria: no sexual activity in the previous 48 h and no use of vaginal medication or washing in the previous 48 h. Women who were menstruating or pregnant were excluded.

The study population consisted of gynecological outpatients and asymptomatic women. They were devided into 6 age groups in the study.

All the samples and their clinical information were obtained following protocols approved by the ethics committees of the local hospitals, and the written informed consent was signed by all the participants.

### Specimen collection

Cervical cell scrapings were collected with a cytobrush included in the testing kit provided by the company. The cervical samples were kept in a sample transport medium for HPV detection and a cell preservation solution for cytology testing. All samples were shipped to the lab at 4 °C and the test was performed within 48 h. The specimens were kept at room temperature for no longer than 12 h before examination. Specific HPV genotypes were using the HPV Genoarray test kit(Yaneng Biotechnology Limited Corp. Shenzhen, China), a PCR-based hybridization gene chip assay.

### Genotype-specific test

HPV detection and genotyping were performed using a commercial HPV Genotyping Kit for 23 HPV types (Yaneng Biotechnology Limited Corp. Shenzhen, China), including 17 HR-HPV types (16, 18, 31, 33, 35, 39, 45, 51, 52, 53, 56, 58, 59, 66, 68, 73 and 82) and 6 LR-HPV types (6, 11, 42, 43, 81 and 83). This kit has been approved by the China Food and Drug Administration (CFDA). The protocol, including DNA extraction, polymerase chain reaction (PCR) and reverse dot blot hybridization (RDB), are described specifically below. All the experimental procedures were strictly followed the manufacturer’s instructions. DNA extraction, PCR amplification and genotypes detection were conducted as follows.

Firstly, the liquid-based cytology specimen was centrifuged in an Eppendorf tube. After the supernatant was discarded, DNA was extracted. DNA concentration and purity (OD260/OD280 between 1.6–1.8) were examined using a spectrophotometer to determine. Next, PCR was carried out. Amplification was started with initial denaturation at 95 °C for 5 min, followed by 5 cycles of denaturation at 95 °C for 30 s, annealing at 58 °C for 30 s and elongation at 72 °C for 30 s. An additional 35 cycles were conducted, carried out including denaturation at 95 °C for 30 s, annealing at 55 °C for 30 s, and extension at 72 °C for 30 s and a final extension at 72 °C for 3 min. The kit employs DNA amplification to detect HPV positivity in a microarray format, utilizing a nylon membrane onto which HPV genotype-specific oligonucleotide probes secured have been immobilized to simultaneously identify 23 HPV genotypes. The blue spots on the strip test strip, indicating a positive result, could be discerned by the naked eye.

The screened population was divided into 6 groups based on their ages. The T1 group indicates age ≤ 20; the T2 group indicates 20 < age ≤ 30; the T3 group indicates 30 < age ≤ 40; the T4 group indicates 40 < age ≤ 50; the T5 group indicates 50 < age ≤ 60; and the T6 group indicates age > 60.

### Statistical analysis

All statistical analyses were performed by using SPSS 19.0 statistical package for Windows. A X^2^ test was utilized to evaluate the significance of difference between designated groups. All tests were two-sided and interpreted as being significant at *p* ≤ 0.05*.*

## Results

### Characteristics of the study participants

In this study, a total of 62,317 women from three cities in Jiangsu Province met the participation criteria, and underwent an outpatient gynecological examination. The mean age of the participants was 38.25, and the age ranged from 16 to 92 years (Fig. 1). Of the 62,317 subjects, 16,775 women tested positive for HPV infection, with an overall HPV infection rate of 26.92% (16,775/62,317), and an HR-HPV positive rate of 22.96% (14,306/62,317). Thus, 85.28% of infections were caused by HR-HPV. The most commonly detected HR-HPV genotypes were HPV52 (5.09%, 3170/62,317), followed by HPV16 (5.06%, 3156/62,317), HPV58 (3.14%, 1959/62,317), HPV53 (2.46%, 1530/62,317), HPV51 (2.07%, 1289/62,317) and HPV68 (2.02%, 1258/62,317). The most commonly detected LR-HPV genotypes were: 2.70% HPV81 (1685/62,317), 1.59% HPV43 (991/62,317), 1.58% HPV42 (986/62,317), 1.54% HPV6(959/62,317), 1.15% HPV11 (715/62,317). 0.36% HPV83(222/62,317). The HPV-positive rates in Xuzhou, Nanjing and Suzhou were 24.04, 28.79 and 28.72% respectively (Table 1). The HPV-positive rates are significantly different between the three regions(*p* < 0.05), with a lower prevalence in Xuzhou, and a higher similar prevalence rates in Nanjing and Suzhou. The six most prevalent HR-HPV HPV genotypes in Xuzhou and Nanjing were 52, 16, 58, 53, 51, and 68; however the top six HR-HPV genotypes in Suzhou were 16, 58, 52, 33, 18, and 68. The two most prevalent LR-HPV genotypes in Xuzhou and Nanjing were 81 and 43, but in Suzhou the most two common LR-HPV genotypes were 11 and 6 (see Table 2). Single HPV genotype infection was found to be the most common pattern (64.37%, 10,872/16,889); in Xuzhou single HPV genotype infection occurred at a rate of 65.59% (4273/6515), in Nanjing it was 66.98% (5135/7667), in Suzhou it was 54.08% (1464/2707). The multiple genotype infection rate was 35.63% (6017/16,889), and the most mixed infection included 9 genotypes.

**Table 1:** HPV positive rates in the three regions (*p < 0.05*)

Regions	Samples	Positive	Prevalence
Xuzhou	26,262	6401	24.04%
Nanjing	26,629	7667	28.79%
Suzhou	9426	2707	28.72%
Total	62,317	16,775	26.92%

**Table 2:** 23HPV genotype prevalence rates by regions

HPV genotype	Xuzhou	Prevalence	Nanjing	Prevalence	Suzhou	Prevalence
52	1232	4.69%	1654	6.21%	284	3.01%
16	1218	4.64%	1336	5.02%	602	6.39%
58	676	2.57%	953	3.58%	330	3.50%
53	605	2.30%	863	3.24%	62	0.66%
51	538	2.05%	651	2.44%	100	1.06%
68	525	2.00%	563	2.11%	170	1.80%
56	397	1.51%	554	2.08%	167	1.77%
33	355	1.35%	466	1.75%	249	2.64%
18	370	1.41%	417	1.57%	194	2.06%
59	308	1.17%	355	1.33%	104	1.10%
66	320	1.22%	349	1.31%	92	0.98%
31	233	0.89%	348	1.31%	142	1.51%
39	184	0.70%	322	1.21%	85	0.90%
35	219	0.83%	246	0.92%	130	1.38%
45	97	0.37%	137	0.51%	58	0.62%
82	34	0.13%	56	0.21%	45	0.48%
73	55	0.21%	45	0.17%	39	0.41%
*81*	*764*	*2.91%*	*848*	*3.18%*	*73*	*0.77%*
*83*	*57*	*0.22%*	*72*	*0.27%*	*93*	*0.99%*
*43*	*404*	*1.54%*	*481*	*1.81%*	*106*	*1.12%*
*42*	*392*	*1.49%*	*462*	*1.73%*	*132*	*1.40%*
*6*	*401*	*1.53%*	*246*	*0.92%*	*312*	*3.31%*
*11*	*228*	*0.87%*	*151*	*0.57%*	*336*	*3.56%*

**Fig. 2:**
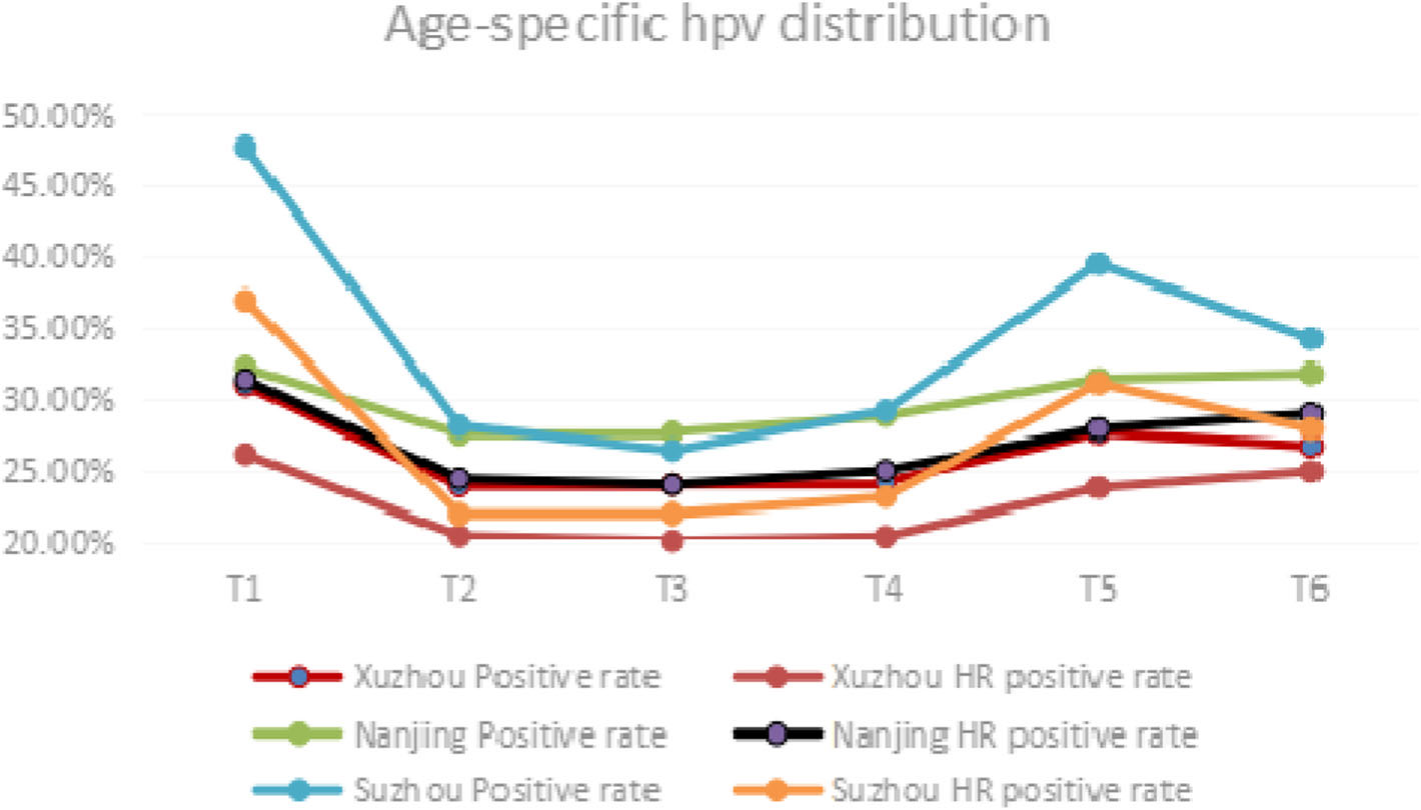
Age-specific HPV positive rate in 3 cities

The HR-HPV infection accounted for 84.77% of the proportion in total HPV infection in Xuzhou, and 87.60% in Nanjing; however, a higher rate (90.91%) of HR-HPV infection occurred in Suzhou (Table 3). Age-specific HPV prevalence presented itself in an U style distribution(Fig. 2), meaning that the two peaks of data points emerged in the T1 group and then in T6 or T5 groups (Table 3). The HR-HPV infection rate exhibited a parallel change. The prevalence of HPV was significantly different between the different age groups and peaked in the ≤20 (T1) year group and the T5 or the T6 groups. Prevalence was high in younger than 20-year-old, it showed a downward trend with increasing age, and another high level of prevalence emerged in the T5 and T6 groups.

**Table 3:** Prevalence in different age groups (*p < 0.05*)

	Xuzhou		Nanjing		Suzhou	
	Positive rate	HR positive rate	Positive rate	HR positive rate	Positive rate	HR positive rate
T1	30.98%	26.16%	32.23%	31.40%	47.73%	36.93%
T2	23.89%	20.44%	27.55%	24.42%	28.13%	21.93%
T3	24.00%	20.07%	27.67%	23.99%	26.42%	21.96%
T4	24.18%	20.37%	28.81%	24.99%	29.18%	23.11%
T5	27.66%	23.78%	31.45%	27.93%	39.46%	31.13%
T6	26.72%	24.94%	31.82%	28.98%	34.23%	27.93%
Total	24.37%	20.66%	28.79%	25.22%	28.72%	22.96%

## Discussion

In the present study, the presence of HPV infection among the females who underwent the gynecological examination in three cities of Jiangsu Province was tested utilizing a commercially available HPV DNA kit. The overall HPV prevalence in Jiangsu province is 26.92%, which is a higher rate than was found in a previous study in Zhejiang (20.54%) [10], and lower that the rate found in Henan (44.5%) [11] and Qingdao (32.2%) [12]. This could be attributed to the difference in the sample population. In the present study, the primary cohorts in the study were sexually active women, and there were fewer subjects in the T1 and T6 groups. Furthermore, people who come to the hospitals typically present in an unhealthy state. All these factors could have influenced the final results of the study. The prevalence features of HPV infection were in congruence with a previous report based in Taizhou [13], which is another city in Jiangsu Province. Both Zhejiang and Jiangsu lie in the southeast of mainland China. The two provinces share some common geographic and socioeconomic characteristics, as well as similarities in the data on HPV infections, such as the prevalent genotypes, the overall infection rate and age-specific prevalence distribution. Irrespective of cervical cytology, HPV52 is the most prevalent high-risk genotype detected in Jiangsu, followed by HPV16 and HPV58. These top three genotypes prevalence rates are consistently with the data generated by Chinese population-specific investigations [14]. HPV16 is an important genotype to consider relating to carcinogenesis, as it was found in 58.70% of invasive cervical cancer. HPV52 and HPV58 showed a much lower correlation with invasive cervical cancer compared to HPV16 [10]. Prophylactic vaccination against HPV, especially HPV16 to help prevent cervical cancer and its precursors, has been very effective [15]. Specifically, HPV16 ranked first in Suzhou and the prevalence reached up to 6.31%; however, HPV52 ranked first in Xuzhou and Nanjing. The differences suggested that HPV prevalence varies within a province, as much as it varies between different geographical regions [16]. HPV18 with an overall prevalence of 1.57%, was not included in the top six HR-HPV genotypes in the present study; this finding differs from the data acquired from the control women of a pooled data of 11 case-control studies from nine countries. Among 1928 women in the control group, HPV types 16, 18, 45, 31, 6, 58, 35, and 33 were the most common infection genotypes [1]. In Suzhou, however, HPV18 ranked the fifth in HR-HPV. Possible causes of this difference could be geographic or economic factors, lifestyle or living standard. The economic factors, lifestyle or living standard may affect the sexual behaviors between persons and the geographic factors may affect the virus itself. Features of HPV prevalence in Suzhou showed more infections come from HPV16 and 18, the prevention of HPV infection in Suzhou warrants more attention. The quadrivalent HPV Vaccine available on the market is theoretically suitable for the women living in Suzhou region, it was shown to be more cost-effective in this region compared to Nanjing and Xuzhou, as this vaccine covers the HPV6 and HPV11 genotypes, which were dominant in LR-HPV infection in Suzhou subjects. Consistent with the age of onset of cervical squamous cell carcinoma, most infections in young women did not become persistent and were characterized as transient infections. Persistent infections in middle-aged women led to cervical lesions. Use of the quadrivalent HPV vaccine Gardasil4 was associated with a decrease in diseases related to vaccine-type HPV infections in women between 25 and 45 years of age, and a high serum antibody level lasted for years [9]; this effect serves as a strong weapon against persistent HPV infections.

Notably, the prevalence of HPV infection was significantly different between age groups [17]. The first peak of HPV infection rate occurred in the younger than 20 year old group; this result could be due to a lack of adaptive immune response and susceptibility to new HPV infection types. The second peak was observed in the T5 or T6 group; this could be partly explained by viral persistence or reactivation of latent HPV due to physiologic and immunologic dysregulation [18] caused by hormone decrease during the menopausal transition.

From the data we collected, single genotype infections were observed more frequently than multiple genotype infections, and made up 54% of the total infections. The most common type of multiple infection was a double infection. HPV16 and HPV52 were the most frequent combination in Xuzhou and Nanjing, but in Suzhou, the most frequent combination was HPV16 and HPV33. The maximum quantity of different genotypes in a multiple infection was 9 HPV genotypes. It has been reported that multiple infections often promote the development of cervical diseases [19], increasing the likelihood of abnormal cytology compared to those with single HPV type infections [20].

In conclusion, we performed a population-based study aimed at detecting HPV prevalence in three representative cities of Jiangsu Province. The age-specific and region-specific infection rates demonstrated significant variance; this finding will provide guidance and increase the accuracy of future vaccination programming. Further study should focus on infection patterns, as demonstrated by the correlation between pathology and infection genotypes. It is important to discover which populations are inclined to persistent versus transient infections, as this understanding is crucial for precise vaccination of the target population.
